# (Benzoato-κ*O*)bis­(1,10-phenanthroline-κ^2^ *N*,*N*′)copper(II) chloride benzoic acid disolvate

**DOI:** 10.1107/S1600536810011487

**Published:** 2010-04-02

**Authors:** Wen-Xiang Huang, Bin-Bin Liu, Jian-Li Lin

**Affiliations:** a State Key Laboratory Base of Novel Functional Materials and Preparation Science, Center of Applied Solid State Chemistry Research, Ningbo University, Ningbo, Zhejiang 315211, People’s Republic of China

## Abstract

In the title complex, [Cu(C_7_H_5_O_2_)(C_12_H_8_N_2_)_2_]Cl·2C_6_H_5_CO­OH, the Cu^II^ ion is coordinated by one carboxyl­ate O atom from a benzoate anion and four N atoms from two phenantroline ligands in a distorted five-coordinate trigonal-bipyramidal CuON_4_ chromophore. The Cu^2+^ and the Cl^−^ ion are imposed by a twofold rotation axiss which also bisects the equally disordered benzoate anion. In the crystal, the mol­ecules are assembled into chains along [010] by C—H⋯Cl, O—H⋯Cl and C—H⋯O hydrogen-bonding inter­actions. The resulting chains are further connected into two-dimensional supra­molecular layers parallel to [100] by inter­chain π⋯π stacking inter­actions [centroid–centroid distance = 3.823 (5) Å] between the phenanthroline ligands and the benzoic acid mol­ecules, and by C—H⋯O hydrogen-bonding inter­actions. Strong π⋯π stacking inter­actions between adjacent phenantroline ligands [3.548 (4) Å] assemble the layers into a three-dimensional supra­molecular architecture.

## Related literature

For copper–aromatic acid coordination polymers, see: Li *et al.* (2006 ▶); Devereux *et al.* (2007 ▶). For related structures, see: Mao *et al.* (2001 ▶). For the τ parameter, see: Addison *et al.* (1984 ▶).
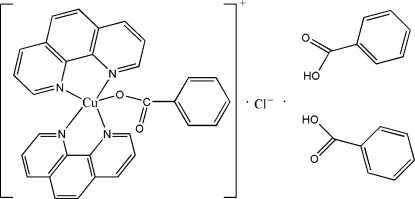


## Experimental

### 

#### Crystal data

[Cu(C_7_H_5_O_2_)(C_12_H_8_N_2_)_2_]Cl·2C_7_H_6_O_2_
*M* *_r_* = 824.74
Monoclinic, 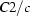

*a* = 16.724 (3) Å
*b* = 19.288 (4) Å
*c* = 13.295 (3) Å
β = 113.86 (3)°
*V* = 3922.1 (14) Å^3^
*Z* = 4
Mo *K*α radiation
μ = 0.68 mm^−1^
*T* = 293 K
0.35 × 0.31 × 0.28 mm

#### Data collection

Rigaku R-AXIS RAPID diffractometer
Absorption correction: multi-scan (*ABSCOR*; Higashi, 1995 ▶) *T*_min_ = 0.710, *T*_max_ = 0.750
15193 measured reflections
3449 independent reflections
2623 reflections with *I* > 2σ(*I*)
*R*_int_ = 0.029

#### Refinement

*R*[*F* ^2^ > 2σ(*F* ^2^)] = 0.038
*wR*(*F* ^2^) = 0.101
*S* = 1.08
3449 reflections
286 parameters
H atoms treated by a mixture of independent and constrained refinement
Δρ_max_ = 0.33 e Å^−3^
Δρ_min_ = −0.45 e Å^−3^

### 

Data collection: *RAPID-AUTO* (Rigaku, 1998 ▶); cell refinement: *RAPID-AUTO*; data reduction: *CrystalStructure* (Rigaku/MSC, 2004 ▶); program(s) used to solve structure: *SHELXS97* (Sheldrick, 2008 ▶); program(s) used to refine structure: *SHELXL97* (Sheldrick, 2008 ▶); molecular graphics: *ORTEPII* (Johnson, 1976 ▶); software used to prepare material for publication: *SHELXL97*.

## Supplementary Material

Crystal structure: contains datablocks global, I. DOI: 10.1107/S1600536810011487/zq2032sup1.cif

Structure factors: contains datablocks I. DOI: 10.1107/S1600536810011487/zq2032Isup2.hkl

Additional supplementary materials:  crystallographic information; 3D view; checkCIF report

## Figures and Tables

**Table 1:** Hydrogen-bond geometry (Å, °)

*D*—H⋯*A*	*D*—H	H⋯*A*	*D*⋯*A*	*D*—H⋯*A*
C5—H5*A*⋯Cl	0.96	2.94	3.728 (4)	140
O3—H31⋯Cl	0.85 (4)	2.20 (4)	3.051 (3)	177 (4)
O3—H31⋯Cl^i^	0.85 (4)	2.20 (4)	3.051 (3)	177 (4)
C24—H24*A*⋯O4^ii^	0.93	2.49	3.355 (5)	155
C8—H8*A*⋯O3^iii^	0.93	2.47	3.307 (4)	149
C10—H10*A*⋯O1^iv^	0.93	2.53	3.275 (7)	138
C12—H12*A*⋯O1^iv^	0.93	2.30	3.106 (7)	146

Symmetry codes: (i) 
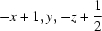
; (ii) 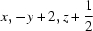; (iii) 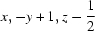; (iv) 
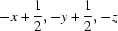
.

**Fig. 1.**
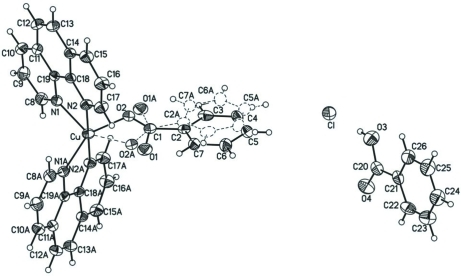
ORTEP view of the title compound. The displacement ellipsoids are drawn at the 20% probability level.

**Fig. 2.**
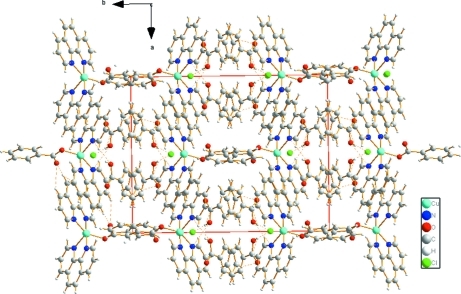
The three-dimensional structure of the title complex through π···π stacking and hydrogen bond interactions.

## supplementary crystallographic information

### Comment

Over the past decades, vast efforts have been dedicated to rational design and synthesis of copper-aromatic-acid coordination polymers, due to their potential applications in medicine, electronics, magnetism, catalysis, gas storage, etc··· It is well known that aromatic carboxylic acids, such as *p*–phthalic acid (Li *et al.*, 2006) and salicylic acid (Devereux *et al.*, 2007), were used to construct coordination polymers with copper salts and exhibited interesting electrochemical properties. In the present contribution, we report a new copper coordination complex, [Cu(phen)_2_(C_6_H_5_COO)].2(C_6_H_5_COOH).Cl, resulting from self-assembly of Cu^II^ ions, phenanthroline ligands and benzoic acid molecules.

The crystal structure of the title complex consists of [Cu(phen)_2_(C_6_H_5_COO)]^+^cations, free benzoic acid molecules and uncoordinated Cl^–^ anions in a ratio 1:2:1. The Cu^II^ ion is coordinated by one carboxylate O atom from a benzoate anion and four N atoms from two phenantroline ligands to complete a distorted five–coordinate trigonal bipyramidal CuON_4_ chromophore. The equatorial positions of the Cu^II^ ion are occupied by one O atom and two N atoms from different phen molecules, and the axial ones by the other two N atoms. The Addison's τ value of 0.53 (τ = 0 for an ideal square pyramid and τ = 1 for an ideal trigonal bipyramid) speaks for a trigonal bipyramid character with a '3+2' coordination type (Addison *et al.*, 1984), which is similar to that of Cu atom in the literature (Mao *et al.*, 2001). The dihedral angle between the benzene ring plane and the carboxylate plane of the coordinated benzoic ion is 14.4 (1)°, which is larger than the dihedral angle in the free benzoic acid molecule (6.5 (6)°). In addition, the Cu^II^ ions and the benzoate ligands are crystallographically imposed by 2–fold rotation axes. The molecules are assembled into one-dimensional chains along [010] direction through hydrogen bonds interactions (C5–H5A···Cl, O3–H3A···Cl, C24–H24A···O4, C8–H8A···O3). The resulting chains are further connected into two-dimensional supramolecular layers parallel to [100] by interchain π···π stacking interactions (3.823 (5) Å) between the phenantroline ligands and the molecular benzoic acid, and by hydrogen bonding interactions (C10–H10A···O1, C12–H12A···O1). Furthermore, on the basis of strong π···π stacking interactions between interlayer adjacent phenantroline ligands (3.548 (4) Å), the layers are assembled into a three-dimensional supramolecular architecture.

### Experimental

Dropwise addition of 2.0 mL (1.0 *M*) NaOH to a stirred aqueous solution of 0.1708 g (1.001 mmol) CuCl_2_.H_2_O in 10.0 mL H_2_O afforded a blue precipitate, which was separated by centrifugation and washed with distilled water for 5 times. The gathered precipitate was then transferred into a solution of benzoic acid (0.2448 g, 2.0049 mmol) and 1,10-phenanthroline (0.1986 g, 1.002 mmol) in a mixed solvent composed of 10.0 mL H_2_O and 10.0 mL ethanol to yield a blue suspension. The mixture was then stirred for further 30 min. After filtration, the filtrate was kept at room temperature and afforded a small amount of blue crystalline blocks after 20 days.

### Refinement

H atoms bonded to C atoms were placed in geometrically calculated positions and were refined using a riding model, with *U*_iso_(H) = 1.2 *U*_eq_(C). The H atom attached to O3 was found in a difference Fourier map and was refined using a riding model, with the O—H bond distance fixed as initially found and with *U*_iso_(H) value set at 1.2 *U*_eq_(O).

### Crystal data

**Table:**

[Cu(C_7_H_5_O_2_)(C_12_H_8_N_2_)_2_]Cl·2C_7_H_6_O_2_	*F*(000) = 1700
*M**_r_* = 824.74	*D*_x_ = 1.397 Mg m^−^^3^
Monoclinic, *C*2/*c*	Mo *K*α radiation, λ = 0.71073 Å
Hall symbol: -C 2yc	Cell parameters from 15193 reflections
*a* = 16.724 (3) Å	θ = 3.2–25.0°
*b* = 19.288 (4) Å	µ = 0.68 mm^−^^1^
*c* = 13.295 (3) Å	*T* = 293 K
β = 113.86 (3)°	Block, blue
*V* = 3922.1 (14) Å^3^	0.35 × 0.31 × 0.28 mm
*Z* = 4	

### Data collection

**Table:**

Rigaku R-AXIS RAPID diffractometer	3449 independent reflections
Radiation source: fine-focus sealed tube	2623 reflections with *I* > 2σ(*I*)
graphite	*R*_int_ = 0.029
Detector resolution: 0 pixels mm^-1^	θ_max_ = 25.0°, θ_min_ = 3.2°
ω scans	*h* = −19→17
Absorption correction: multi-scan (*ABSCOR*; Higashi, 1995)	*k* = −22→22
*T*_min_ = 0.710, *T*_max_ = 0.750	*l* = −15→15
15193 measured reflections	

### Refinement

**Table:**

Refinement on *F*^2^	Primary atom site location: structure-invariant direct methods
Least-squares matrix: full	Secondary atom site location: difference Fourier map
*R*[*F*^2^ > 2σ(*F*^2^)] = 0.038	Hydrogen site location: inferred from neighbouring sites
*wR*(*F*^2^) = 0.101	H atoms treated by a mixture of independent and constrained refinement
*S* = 1.08	*w* = 1/[σ^2^(*F*_o_^2^) + (0.0393*P*)^2^ + 3.5087*P*] where *P* = (*F*_o_^2^ + 2*F*_c_^2^)/3
3449 reflections	(Δ/σ)_max_ = 0.001
286 parameters	Δρ_max_ = 0.33 e Å^−^^3^
0 restraints	Δρ_min_ = −0.45 e Å^−^^3^

### Special details

**Table:**

Geometry. All esds (except the esd in the dihedral angle between two l.s. planes) are estimated using the full covariance matrix. The cell esds are taken into account individually in the estimation of esds in distances, angles and torsion angles; correlations between esds in cell parameters are only used when they are defined by crystal symmetry. An approximate (isotropic) treatment of cell esds is used for estimating esds involving l.s. planes.
Refinement. Refinement of *F*^2^ against ALL reflections. The weighted *R*-factor wR and goodness of fit *S* are based on *F*^2^, conventional *R*-factors *R* are based on *F*, with *F* set to zero for negative *F*^2^. The threshold expression of *F*^2^ > σ(*F*^2^) is used only for calculating *R*-factors(gt) etc. and is not relevant to the choice of reflections for refinement. *R*-factors based on *F*^2^ are statistically about twice as large as those based on *F*, and *R*- factors based on ALL data will be even larger.

### Fractional atomic coordinates and isotropic or equivalent isotropic displacement parameters (Å^2^)

**Table:**

	*x*	*y*	*z*	*U*_iso_*/*U*_eq_	Occ. (<1)
Cu	0.5000	0.26282 (3)	0.2500	0.05651 (17)	
N1	0.40747 (13)	0.19982 (11)	0.12871 (18)	0.0554 (5)	
N2	0.40438 (13)	0.26263 (12)	0.30525 (18)	0.0565 (5)	
O1	0.4521 (4)	0.3849 (3)	0.1410 (5)	0.090 (2)	0.50
O2	0.5351 (3)	0.3568 (3)	0.3129 (5)	0.0600 (12)	0.50
C1	0.5000	0.4012 (3)	0.2500	0.0678 (11)	
C2	0.5084 (4)	0.47718 (18)	0.2698 (3)	0.0556 (17)	0.50
C3	0.4829 (4)	0.5284 (3)	0.1894 (2)	0.0788 (19)	0.50
H3A	0.4598	0.5159	0.1131	0.095*	0.50
C4	0.4907 (5)	0.5979 (2)	0.2196 (3)	0.075 (3)	0.50
H4A	0.4731	0.6333	0.1641	0.090*	0.50
C5	0.5240 (5)	0.61615 (16)	0.3302 (4)	0.084 (3)	0.50
H5A	0.5295	0.6641	0.3511	0.100*	0.50
C6	0.5495 (3)	0.5649 (2)	0.4106 (3)	0.0794 (18)	0.50
H6A	0.5726	0.5775	0.4869	0.095*	0.50
C7	0.5417 (3)	0.49540 (19)	0.3803 (3)	0.0639 (15)	0.50
H7A	0.5593	0.4600	0.4358	0.077*	0.50
C8	0.4095 (2)	0.16743 (16)	0.0414 (2)	0.0694 (8)	
H8A	0.4614	0.1678	0.0313	0.083*	
C9	0.3373 (2)	0.13300 (16)	−0.0355 (3)	0.0791 (9)	
H9A	0.3417	0.1106	−0.0950	0.095*	
C10	0.2608 (2)	0.13242 (17)	−0.0231 (3)	0.0794 (9)	
H10A	0.2123	0.1097	−0.0742	0.095*	
C11	0.25483 (17)	0.16598 (14)	0.0667 (2)	0.0620 (7)	
C12	0.17667 (18)	0.16982 (17)	0.0868 (3)	0.0790 (9)	
H12A	0.1260	0.1485	0.0379	0.095*	
C13	0.17515 (18)	0.20324 (17)	0.1738 (3)	0.0773 (9)	
H13A	0.1231	0.2055	0.1835	0.093*	
C14	0.25144 (16)	0.23560 (14)	0.2524 (2)	0.0596 (7)	
C15	0.25428 (19)	0.27038 (17)	0.3459 (3)	0.0726 (8)	
H15A	0.2041	0.2741	0.3596	0.087*	
C16	0.3303 (2)	0.29866 (18)	0.4164 (3)	0.0773 (9)	
H16A	0.3330	0.3211	0.4796	0.093*	
C17	0.40467 (19)	0.29405 (17)	0.3938 (3)	0.0728 (8)	
H17A	0.4565	0.3138	0.4430	0.087*	
C18	0.32904 (15)	0.23281 (13)	0.2352 (2)	0.0502 (6)	
C19	0.33051 (15)	0.19836 (13)	0.1411 (2)	0.0509 (6)	
C20	0.6442 (2)	0.89663 (16)	0.5209 (3)	0.0723 (8)	
C21	0.6652 (2)	0.94046 (15)	0.6202 (3)	0.0673 (8)	
C22	0.7514 (2)	0.9571 (2)	0.6830 (3)	0.0868 (10)	
H22A	0.7949	0.9405	0.6626	0.104*	
C23	0.7738 (3)	0.9976 (2)	0.7744 (3)	0.1056 (12)	
H23A	0.8322	1.0083	0.8157	0.127*	
C24	0.7114 (3)	1.0223 (2)	0.8054 (4)	0.1033 (12)	
H24A	0.7268	1.0498	0.8680	0.124*	
C25	0.6259 (3)	1.0067 (2)	0.7445 (4)	0.1094 (14)	
H25A	0.5833	1.0236	0.7662	0.131*	
C26	0.6014 (2)	0.96621 (18)	0.6513 (3)	0.0901 (11)	
H26A	0.5427	0.9564	0.6100	0.108*	
Cl	0.5000	0.80270 (7)	0.2500	0.0916 (4)	
O4	0.69924 (15)	0.87024 (14)	0.4972 (2)	0.1004 (8)	
O3	0.55968 (17)	0.88975 (14)	0.4593 (2)	0.0977 (8)	
H31	0.544 (3)	0.867 (2)	0.400 (3)	0.117*	

### Atomic displacement parameters (Å^2^)

**Table:**

	*U*^11^	*U*^22^	*U*^33^	*U*^12^	*U*^13^	*U*^23^
Cu	0.0442 (3)	0.0686 (3)	0.0647 (3)	0.000	0.0303 (2)	0.000
N1	0.0514 (12)	0.0577 (13)	0.0631 (13)	0.0028 (10)	0.0293 (11)	−0.0023 (11)
N2	0.0457 (11)	0.0689 (14)	0.0605 (13)	−0.0025 (10)	0.0274 (11)	−0.0060 (11)
O1	0.074 (3)	0.099 (5)	0.066 (4)	0.006 (3)	−0.003 (3)	−0.021 (3)
O2	0.048 (3)	0.066 (3)	0.065 (3)	−0.003 (2)	0.021 (3)	−0.001 (2)
C1	0.043 (2)	0.069 (3)	0.090 (4)	0.000	0.025 (2)	0.000
C2	0.036 (3)	0.071 (3)	0.057 (5)	0.001 (4)	0.015 (4)	0.008 (3)
C3	0.051 (4)	0.108 (6)	0.068 (4)	0.009 (4)	0.014 (3)	0.018 (4)
C4	0.066 (5)	0.059 (4)	0.099 (10)	0.010 (5)	0.031 (7)	0.030 (4)
C5	0.066 (5)	0.062 (4)	0.118 (8)	0.009 (4)	0.033 (6)	−0.016 (5)
C6	0.086 (4)	0.067 (4)	0.077 (4)	0.000 (3)	0.025 (4)	−0.008 (4)
C7	0.065 (4)	0.060 (4)	0.062 (4)	0.000 (3)	0.020 (3)	−0.004 (3)
C8	0.0703 (18)	0.071 (2)	0.077 (2)	0.0054 (16)	0.0401 (17)	−0.0067 (16)
C9	0.088 (2)	0.071 (2)	0.075 (2)	−0.0002 (18)	0.0290 (18)	−0.0246 (17)
C10	0.0640 (19)	0.075 (2)	0.085 (2)	−0.0066 (16)	0.0146 (17)	−0.0168 (18)
C11	0.0550 (16)	0.0535 (16)	0.0726 (19)	−0.0011 (13)	0.0207 (14)	−0.0038 (14)
C12	0.0483 (16)	0.078 (2)	0.105 (3)	−0.0131 (15)	0.0248 (17)	−0.009 (2)
C13	0.0491 (16)	0.081 (2)	0.108 (3)	−0.0054 (15)	0.0388 (18)	0.000 (2)
C14	0.0462 (14)	0.0615 (17)	0.0778 (18)	0.0016 (13)	0.0322 (14)	0.0083 (15)
C15	0.0616 (18)	0.086 (2)	0.090 (2)	0.0066 (16)	0.0514 (18)	0.0042 (18)
C16	0.071 (2)	0.097 (2)	0.080 (2)	0.0014 (18)	0.0474 (18)	−0.0120 (18)
C17	0.0627 (17)	0.093 (2)	0.0726 (19)	−0.0080 (16)	0.0373 (16)	−0.0174 (17)
C18	0.0423 (13)	0.0502 (14)	0.0619 (15)	0.0033 (11)	0.0248 (12)	0.0061 (13)
C19	0.0450 (13)	0.0464 (15)	0.0629 (16)	0.0042 (11)	0.0237 (13)	0.0044 (12)
C20	0.080 (2)	0.0680 (19)	0.093 (2)	0.0097 (16)	0.061 (2)	0.0170 (17)
C21	0.083 (2)	0.0550 (17)	0.088 (2)	0.0119 (15)	0.0592 (19)	0.0123 (15)
C22	0.073 (2)	0.106 (3)	0.093 (3)	0.0252 (19)	0.044 (2)	0.014 (2)
C23	0.089 (3)	0.132 (4)	0.093 (3)	0.011 (2)	0.034 (2)	−0.005 (3)
C24	0.115 (3)	0.106 (3)	0.105 (3)	−0.002 (3)	0.061 (3)	−0.014 (2)
C25	0.115 (3)	0.105 (3)	0.146 (4)	−0.003 (3)	0.093 (3)	−0.031 (3)
C26	0.084 (2)	0.085 (2)	0.129 (3)	−0.0038 (18)	0.072 (2)	−0.016 (2)
Cl	0.0794 (8)	0.0839 (8)	0.1148 (10)	0.000	0.0425 (7)	0.000
O4	0.0964 (16)	0.121 (2)	0.1100 (18)	0.0257 (15)	0.0688 (15)	−0.0062 (15)
O3	0.0869 (17)	0.113 (2)	0.118 (2)	−0.0093 (14)	0.0673 (17)	−0.0255 (16)

### Geometric parameters (Å, °)

**Table:**

Cu—O2	1.984 (5)	C11—C19	1.399 (4)
Cu—O2^i^	1.984 (5)	C11—C12	1.438 (4)
Cu—N2^i^	2.012 (2)	C12—C13	1.334 (4)
Cu—N2	2.012 (2)	C12—H12A	0.9300
Cu—N1	2.110 (2)	C13—C14	1.425 (4)
Cu—N1^i^	2.110 (2)	C13—H13A	0.9300
N1—C8	1.330 (3)	C14—C15	1.396 (4)
N1—C19	1.362 (3)	C14—C18	1.407 (3)
N2—C17	1.322 (3)	C15—C16	1.352 (4)
N2—C18	1.355 (3)	C15—H15A	0.9300
O1—C1	1.378 (6)	C16—C17	1.395 (4)
O2—C1	1.174 (6)	C16—H16A	0.9300
C1—C2	1.486 (6)	C17—H17A	0.9300
C2—C3	1.3900	C18—C19	1.425 (3)
C2—C7	1.3900	C20—O4	1.200 (3)
C3—C4	1.3900	C20—O3	1.323 (4)
C3—H3A	0.9600	C20—C21	1.485 (4)
C4—C5	1.3900	C21—C22	1.380 (5)
C4—H4A	0.9600	C21—C26	1.384 (4)
C5—C6	1.3900	C22—C23	1.363 (5)
C5—H5A	0.9601	C22—H22A	0.9300
C6—C7	1.3900	C23—C24	1.355 (5)
C6—H6A	0.9599	C23—H23A	0.9300
C7—H7A	0.9601	C24—C25	1.361 (5)
C8—C9	1.395 (4)	C24—H24A	0.9300
C8—H8A	0.9300	C25—C26	1.379 (5)
C9—C10	1.356 (4)	C25—H25A	0.9300
C9—H9A	0.9300	C26—H26A	0.9300
C10—C11	1.395 (4)	Cl—Cl^i^	0.000 (3)
C10—H10A	0.9300	O3—H31	0.85 (4)
			
O2—Cu—O2^i^	47.8 (3)	C11—C10—H10A	120.1
O2—Cu—N2^i^	90.69 (16)	C10—C11—C19	117.1 (3)
O2^i^—Cu—N2^i^	89.49 (16)	C10—C11—C12	124.5 (3)
O2—Cu—N2	89.49 (16)	C19—C11—C12	118.4 (3)
O2^i^—Cu—N2	90.69 (16)	C13—C12—C11	121.6 (3)
N2^i^—Cu—N2	179.80 (13)	C13—C12—H12A	119.2
O2—Cu—N1	148.10 (18)	C11—C12—H12A	119.2
O2^i^—Cu—N1	101.76 (18)	C12—C13—C14	121.5 (3)
N2^i^—Cu—N1	99.53 (8)	C12—C13—H13A	119.3
N2—Cu—N1	80.35 (8)	C14—C13—H13A	119.3
O2—Cu—N1^i^	101.76 (18)	C15—C14—C18	117.5 (3)
O2^i^—Cu—N1^i^	148.10 (18)	C15—C14—C13	124.1 (3)
N2^i^—Cu—N1^i^	80.35 (8)	C18—C14—C13	118.4 (3)
N2—Cu—N1^i^	99.53 (8)	C16—C15—C14	119.7 (2)
N1—Cu—N1^i^	109.69 (12)	C16—C15—H15A	120.2
C8—N1—C19	117.0 (2)	C14—C15—H15A	120.2
C8—N1—Cu	131.86 (18)	C15—C16—C17	119.7 (3)
C19—N1—Cu	110.87 (16)	C15—C16—H16A	120.2
C17—N2—C18	118.3 (2)	C17—C16—H16A	120.2
C17—N2—Cu	127.38 (19)	N2—C17—C16	122.6 (3)
C18—N2—Cu	113.88 (16)	N2—C17—H17A	118.7
C1—O2—Cu	112.8 (4)	C16—C17—H17A	118.7
O2—C1—O1	119.6 (6)	N2—C18—C14	122.3 (2)
O2—C1—C2	127.5 (4)	N2—C18—C19	117.4 (2)
O1—C1—C2	112.6 (4)	C14—C18—C19	120.3 (2)
C3—C2—C7	120.0	N1—C19—C11	123.5 (2)
C3—C2—C1	126.0 (3)	N1—C19—C18	116.7 (2)
C7—C2—C1	113.9 (3)	C11—C19—C18	119.8 (2)
C4—C3—C2	120.0	O4—C20—O3	122.4 (3)
C4—C3—H3A	120.0	O4—C20—C21	122.9 (3)
C2—C3—H3A	120.0	O3—C20—C21	114.6 (3)
C3—C4—C5	120.0	C22—C21—C26	118.6 (3)
C3—C4—H4A	120.0	C22—C21—C20	119.0 (3)
C5—C4—H4A	120.0	C26—C21—C20	122.5 (3)
C4—C5—C6	120.0	C23—C22—C21	121.1 (3)
C4—C5—H5A	120.0	C23—C22—H22A	119.5
C6—C5—H5A	120.0	C21—C22—H22A	119.5
C7—C6—C5	120.0	C24—C23—C22	120.3 (4)
C7—C6—H6A	120.0	C24—C23—H23A	119.8
C5—C6—H6A	120.0	C22—C23—H23A	119.8
C6—C7—C2	120.0	C23—C24—C25	119.6 (4)
C6—C7—H7A	120.0	C23—C24—H24A	120.2
C2—C7—H7A	120.0	C25—C24—H24A	120.2
N1—C8—C9	122.9 (3)	C24—C25—C26	121.3 (3)
N1—C8—H8A	118.5	C24—C25—H25A	119.4
C9—C8—H8A	118.5	C26—C25—H25A	119.4
C10—C9—C8	119.6 (3)	C25—C26—C21	119.2 (4)
C10—C9—H9A	120.2	C25—C26—H26A	120.4
C8—C9—H9A	120.2	C21—C26—H26A	120.4
C9—C10—C11	119.9 (3)	C20—O3—H31	119 (3)
C9—C10—H10A	120.1		

Symmetry codes: (i) −*x*+1, *y*, −*z*+1/2.

### Hydrogen-bond geometry (Å, °)

**Table:**

*D*—H···*A*	*D*—H	H···*A*	*D*···*A*	*D*—H···*A*
C5—H5A···Cl	0.96	2.94	3.728 (4)	140
O3—H31···Cl	0.85 (4)	2.20 (4)	3.051 (3)	177 (4)
O3—H31···Cl^i^	0.85 (4)	2.20 (4)	3.051 (3)	177 (4)
C24—H24A···O4^ii^	0.93	2.49	3.355 (5)	155
C8—H8A···O3^iii^	0.93	2.47	3.307 (4)	149
C10—H10A···O1^iv^	0.93	2.53	3.275 (7)	138
C12—H12A···O1^iv^	0.93	2.30	3.106 (7)	146

Symmetry codes: (i) −*x*+1, *y*, −*z*+1/2; (ii) *x*, −*y*+2, *z*+1/2; (iii) *x*, −*y*+1, *z*−1/2; (iv) −*x*+1/2, −*y*+1/2, −*z*.
